# Exploring the role of dietitians in mental health services and the perceived barriers and enablers to service delivery: A cross‐sectional study

**DOI:** 10.1111/jhn.13203

**Published:** 2023-07-12

**Authors:** Scott B. Teasdale, Elise Tripodi, Alexandra Harman, Janice Plain, Tracy L. Burrows

**Affiliations:** ^1^ Mindgardens Neuroscience Network Kensington NSW Australia; ^2^ Discipline of Psychiatry and Mental Health, Faculty of Medicine University of New South Wales Kensington NSW Australia; ^3^ Justice Health and Forensic Mental Health Network Malabar NSW Australia; ^4^ Department of Nutrition and Dietetics Illawarra Shoalhaven Local Health District Wollongong NSW Australia; ^5^ School of Health Sciences, College of Health, Medicine and Wellbeing University of Newcastle Newcastle NSW Australia; ^6^ Food and Nutrition Program Hunter Medical Research Institute New Lambton NSW Australia

**Keywords:** barriers, dietitian, enablers, mental health, mental illness, service provision

## Abstract

**Background:**

Mental health is a rapidly evolving area of practice for dietitians. The role of dietitians in supporting the physical health of consumers experiencing mental illness is becoming more widely recognised given the importance of lifestyle interventions for physical health. The present study aimed to explore the dietitian role in mental health services as well as identify barriers and enablers to service delivery.

**Methods:**

This was a cross‐sectional survey of dietitians currently employed in any capacity in public and private mental health services. An online survey comprised of questions pertaining to four domains, including demographics, role and service provision, experience and supervision, barriers/challenges and drivers/enablers was completed and included closed and open‐ended responses.

**Results:**

In total, 48 responses were included. The mean ± SD age of respondents was 36.1 ± 10.9 years (range 23–67 years) with the majority working in inpatient settings. The top three tasks respondents reported conducting were individual consultations (*n* = 47; 98%), group programs (*n* = 23; 48%) and multidisciplinary team meetings. Barriers included a lack of awareness from others regarding a dietitian's role in mental health, and a lack of specific tools for nutrition screening. More training, resources and increased evidence base to guide practice would enable better service provision.

**Conclusions:**

The present study provides insights regarding the possible drivers and barriers to effective service provision for dietitians working in mental health services focusing on the local contexts of respondents. The findings highlight the importance and value of working collaboratively within a multidisciplinary team.

## Key points

- A survey of Australian dietitians working in mental health identifies major roles undertaken, including individual/group consults and multidisciplinary meetings.
- Barriers reported include a lack of awareness of a dietitian's role in mental health, including their professions capacity to complete roles beyond malnutrition including behaviour change.
- A lack of specific tools was reported for use with mental health populations for nutrition screening/assessment.
- More training and increased evidence base to guide practice would enable better service provision.

## Graphical abstract

A survey of Australian dietitians identifies major roles including individual/group consults and multidisciplinary meetings. The need for advocacy for a dietitian's role in mental health is demonstrated, as well as a lack of specific tools for nutrition screening. More training and increased evidence base to guide practice would enable better service provision.

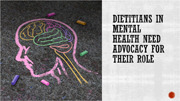

## INTRODUCTION

Mental illness is a rapidly evolving area of practice for dietitians given the high rates of cardiometabolic comorbidities and evolution of dietary modification in the treatment of mental illness. One in five (20%) Australians aged 16–85 years experience a mental illness in any year, and it is estimated that almost half (45%) of Australians will experience some form of mental illness in their lifetime.^1^ With the high prevalence of mental illness affecting Australians, and a surge in rates in the wake of the coronavirus (COVID‐19) pandemic,^2^ every dietitian will encounter consumers experiencing mental illness during their working life.^3^ Individuals experiencing mental illness live 10–15 years less than those without a mental illness, primarily as a result of physical health disparities.^4^ Several lifestyle factors adversely affect the physical health of individuals with mental illness. For example, lower levels of physical activity because of the sedative effect of medications, as well as difficulties accessing and preparing nutritious foods, along with making unhealthy food choices, excessive appetite, poor hydration status, misuse of alcohol and higher levels of food insecurity,^5^, ^6^, ^7^, ^8^ contribute to higher rates of hypertension, dyslipidaemia, diabetes, metabolic syndrome, cardiovascular disease and obesity within this population group.^9^

The role of dietitians in supporting the physical health of people experiencing mental illness is becoming more widely recognised and studied. Role statements developed by professional bodies, such as Dietitians Australia, define the scope of practice for dietitians working in this area. Dietitians are qualified to identify the nutritional needs and diagnose nutrition issues (e.g., risk of malnutrition, disordered food/eating patterns or food insecurity) of individuals, groups and communities with mental illness.^10^ Dietitians in mental health have specific skills to plan, monitor and evaluate evidence‐based dietary interventions at the same time as taking a holistic approach that considers key determinants of health acting on intervention plans.^10^ This includes co‐existing physical health conditions, psychotropic medication side effects and nutrient interactions, as well as behavioural, motivational, social and financial challenges.^10^, ^11^

Dietary intervention is an important component of the treatment for individuals living with mental illness with respect to supporting their physical and mental health. A recent review of 46 systematic reviews,^8^ addressing the effectiveness of dietary interventions for individuals with mental illness, found improved mental and physical health outcomes following dietary intervention such as those focusing on whole of diet approaches. Further research suggests better effects on weight management^12^, ^13^ and clinical outcomes^14^ are achieved when the dietary intervention is led by a dietitian compared to those delivered without dietitian/nutritionist professional involvement. Indeed, the importance of dietitians was acknowledged in a recent systematic review of 25 studies reporting that dietary interventions did not reduce cardiovascular risk markers except when delivered by dietitians.^11^

Although there has been an increased number of mental health dietetic positions created over recent years, the published literature detailing the workforce and describing the landscape of dietitians working in mental health services in Australia and internationally is limited. It is unclear how many dietitians are working in mental health in specific roles and what their level of involvement is. The present study aimed to explore dietitian's role in the mental health facility or service that they currently work in and to gain insight into their perspectives of current dietetic services within their workplace, as well as identify barriers and enablers to service delivery.

## METHODS

### Study design and participants

This cross‐sectional study, conducted from March to April 2022, was approved by The University of Newcastle Human Research Ethics Committee (H‐2021‐0406). Dietitians currently employed in any capacity in public and private mental health services, and hospitals within Australia (i.e., inpatient facilities, community/outpatient services, mental‐health specific non‐government organisations and mental health units within a hospital) were invited to complete an anonymous online survey. The survey took approximately 15 min to complete. Dietitians were recruited via convenience sampling using social media and email through the professional body Dietitians Australia. Additionally, publicly listed dietitians working in private mental health services were contacted. Exclusion criteria included dietitians working in private practice because these dietitians were considered to have different barriers and challenges comapared to those working in public facilities and eating disorder services in that they provide services to consumers under primary health referral pathways (e.g., Medicare) and research has been previously published on their roles.^15^ All data were gathered via the secure REDCap online survey platform (https://www.project-redcap.org) and respondents provided informed consent before completing the survey. Survey completers were invited to enter a prize draw to win one of two shopping vouchers ($100 value).

### Survey measure

The survey consisted of 35–50 fixed response questions depending on the facility they worked in, as well as a further six open‐ended questions that were voluntary. The survey was developed by the research team for this project and modelled on previous research studies that examined dietitian's experiences and perspectives regarding access to and delivery of dietetic services for individuals with type 2 diabetes mellitus^16^ and the role of dietitians in intensive care units.^17^ The survey content is outlined below.

#### Demographics

Questions including the respondent's age, sex, year of graduation, geographical area of practice, years employed in their current role, type of facility respondents they were working in, the inpatient wards or outpatient/community services they provide service to, and the age groups and diagnoses of individuals they provide care to.

#### Role and service provision

This included the type of contract respondents were employed under, level of grading of their position, the FTE worked (full‐time equivalent with 38 h per week classified as full‐time employment with an FTE denoted as 1.0) and the tasks respondents completed in their role. Further questions concerned the role of assistants associated with the dietetic service within their workplace, as well as the nutrition screening, referral and evaluation processes currently in place.

#### Experience and supervision

Questions included level of confidence within their current role and the level of satisfaction with their supervision. These were rated on a five‐point Likert scale, with 1 being lowest and 5 being highest.

#### Barriers/challenges and drivers/enablers

The final part of the survey was voluntary and consisted of six open‐ended questions that asked the respondent their views on the barriers and challenges experienced working in mental health, as well as their views on the drivers and enablers that allow effective service provision within mental health workplace settings.

### Statistical analysis

Descriptive statistics were generated for quantitative data using Stata, version 17 (StataCorp LLC). Data are reported as the mean ± SD for continuous variables and number and percentage for categorical variables. Qualitative data were analysed by two independent researchers, with initial familiarisation of dataset, generating initial codes, searching and reviewing themes, and subsequently naming and defining the themes across the dataset decided by consensus.^18^ Themes and subthemes are illustrated with representative quotations from respondents (R1–R46) that captured the diversity of responses.^18^

## RESULTS

### Sample characteristics

Seventy‐seven individuals completed the screening questions with four individuals deemed ineligible. Of those deemed eligible, 16 individuals did not answer any questions after the screening questions, and a further nine individuals completed <50% of the survey questions and therefore were not included in the current analyses. The final sample size comprised complete survey responses from 48 dietitians, with 46 of these providing responses to the voluntary qualitative questions. The mean ± SD age of respondents was 36.1 ± 10.9 years (range 23–67 years) and most were female (94%) (Table 1). Approximately one‐third of the total sample had graduated within the past 5 years, one‐third had graduated 5–15 years ago and one‐third had graduated ≥ 15 years ago.

**Table 1:** Characteristics of dietitians and their role within the workplace.

Characteristic	*n* (%) or mean ± SD (range)
Gender	
Female	45 (93.7)
Male	2 (4.2)
Prefer not to say	1 (2.1)
Age (years)	36.1 ± 10.9 (23 – 67)
Years as a dietitian	11.1 ± 9.6 (0.1 – 39)
<5	17 (35.4)
5–10	11 (22.9)
10–15	5 (10.4)
>15	15 (31.3)
Years employed in current mental health role	4.4 ± 5.4 (0.1 – 25)
<5	33 (68.8)
5–10	9 (18.8)
>10	6 (12.5)
Type of workplace currently working in^a^	
Public inpatient mental health facility	20 (41.7)
Public community/outpatient mental health service	18 (37.5)
Mental health unit/ward co‐located within a general hospital	5 (10.4)
Public forensic mental health inpatient facility/service	3 (6.3)
Private inpatient mental health facility	8 (16.7)
Private community/outpatient mental health service	1 (2.1)
Other	8 (16.7)
Location of facility/service	
NSW	25 (52.1)
VIC	9 (18.8)
QLD	6 (12.5)
WA	4 (8.3)
SA	4 (8.3)
Age group of consumers^a^	
Children and adolescents (0–16 years)	7 (14.6)
Youth (15–25 years)	25 (52.1)
Adults (16–65 years)	42 (87.5)
Older adults (≥ 65 years)	24 (50.0)
Type of employment contract	
Permanent	32 (66.7)
Temporary/time‐limited contract	14 (29.2)
Casual	2 (4.2)
Level of grading for employment position	
Entry level position (Level 1–2/HP3)	19 (39.6)
Senior clinical/management position (Level 3–4/HP4)	27 (56.3)
Other (not specified)	2 (4.2)

^a^ Cumulative response percentages exceed 100% as respondents were able to select multiple answers.

Abbreviations: NSW, New South Wales; QLD, Queensland; SA, South Australia; VIC, Victoria; WA, Western Australia.

### Workplace settings and consumer/patient/characteristics

Workplaces where respondents were currently employed were located across five Australian states, with the majority being within NSW (52.0%) (Table 1). Seventy‐three percent of respondents (*n* = 35) reported currently working in one mental health facility or service, whereas the remaining reported working across two (*n* = 11) or three (*n* = 2). Across all respondents the average full‐time equivalent for dietitians working in mental health was a 0.57 ± 0.31 FTE role (range 0.1–1.0).

Approximately half of the respondents (52%, *n* = 25) worked in an inpatient setting and twelve respondents worked in an outpatient setting, with the remaining respondents (*n* = 11) working across both settings. The majority of respondents worked predominantly in public facilities or services, with most in either a public inpatient mental health facility (41.7%, *n* = 20) or a public community/outpatient mental health service (37.5%, *n* = 18). A smaller number of respondents worked in ether a private inpatient mental health facility (*n* = 8), a mental health unit/ward co‐located within a general hospital (*n* = 5), a public forensic mental health inpatient facility/service (*n* = 3) or a private community/outpatient mental health service (*n* = 1). A further eight people reported working in another unit that did not fit an aforementioned category such as specialist veteran unit, non‐profit community setting, general rehabilitation/medical unit or clinical research unit.

Dietitians working in inpatient units reported working predominantly within acute adult/older adult units (64.6%), with fewer working in mental health rehabilitation, (27%), long or extended stay (22.9%), children/adolescents (18.8%) or individuals with substance/drug and alcohol use disorders (14.6%). Dietitians working in outpatient services reported working within adults/older adult community health (29.2%), clozapine clinics (16.7%), youth, child and adolescent community health (14.3%), or early psychosis units (6.3%).

**Figure 1:**
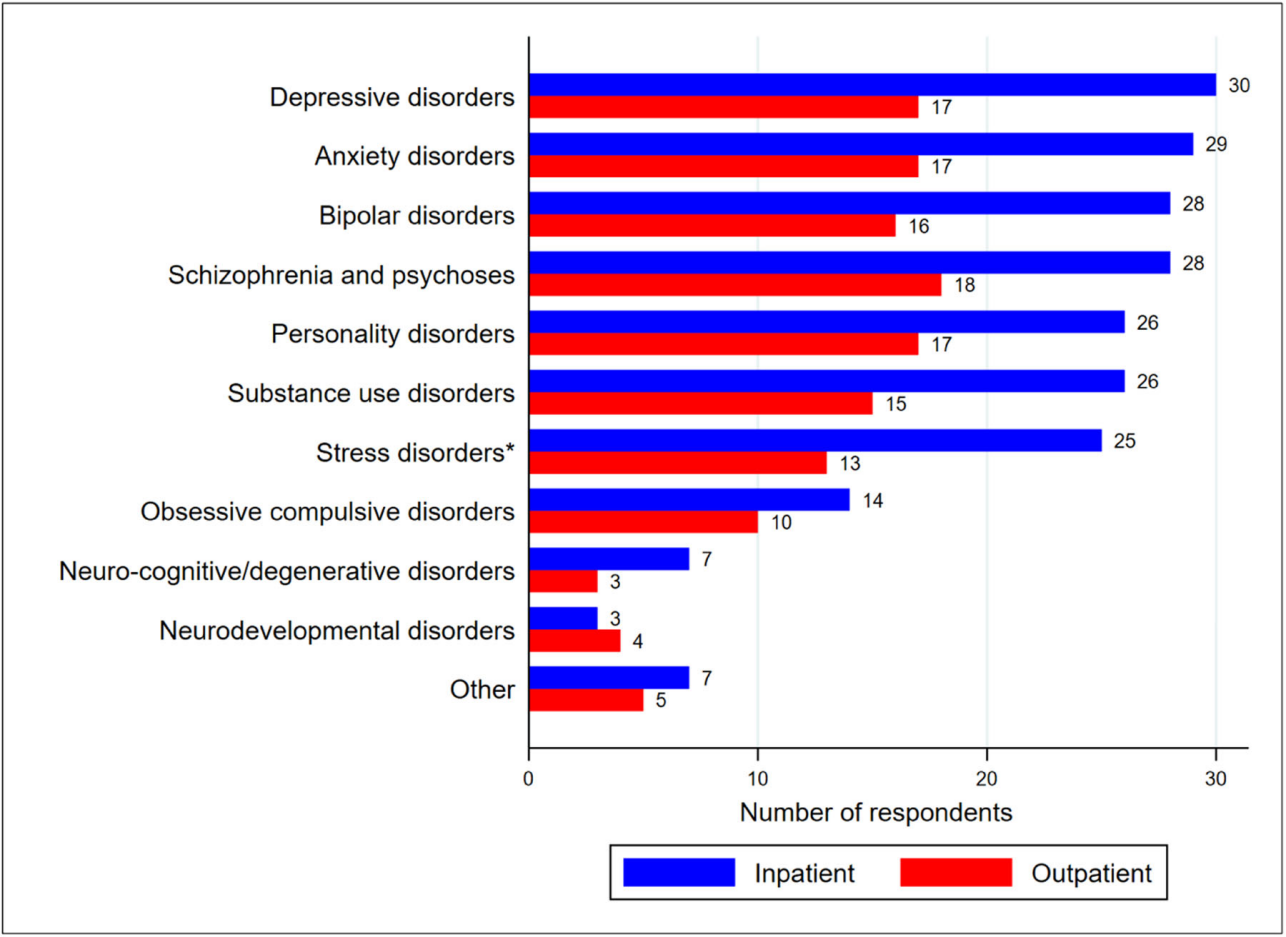
Mental health diagnoses of individuals that dietitian‐respondents (*n* = 48 respondents) provide dietetic care to within their respective inpatient (*n* = 36 workplaces) and outpatient (*n* = 22 workplaces) workplace settings. Respondents were asked ‘What are the common mental health diagnoses of the people you work with?’. Respondents could select more than one response. *Disorders specifically associated with stress, for example, post‐traumatic stress disorder, adjustment disorder, grief disorder.

Of the total sample, approximately three‐quarters (72.9%) of the respondents reported their consumer/patient base encompassed five or more mental health diagnoses. The most common mental health diagnoses (Figure 1) of individuals that respondents provide care to across inpatient and outpatient settings included depressive disorders (79.7%), schizophrenia and related psychoses (78.0%), anxiety disorders (78.0%), bipolar disorders (75.0%), personality disorders (72.9%), substance use disorders (69.5%) and disorders specifically associated with stress (e.g., post‐traumatic stress disorder, adjustment disorder, grief disorder; 64.4%).

### Role of the dietitian services provided in the workplace

The number of years respondents were employed in their current mental health role(s) ranged from 5 weeks to 25 years (mean ± SD: 4.4 ± 5.4 years), with the majority (*n* = 33, 68.8%) employed in their current position for less than 5 years (Table 1). More than half of respondents were employed under a permanent contract (*n* = 32; 67%) or temporary/time‐limited contracts (*n* = 14; 29%). The full‐time equivalent (FTE) across workplaces ranged from 0.1 to 1.0 (mean ± SD: FTE 0.57 ± 0.31). More than half (*n* = 27; 56%) of the respondents were employed in a senior clinical/management position (Level 3–4/HP4 Australian wage rate classification), 19 were employed in an entry level position (Level 1–2/HP3 Australian wage rate classification) and two respondents reported ‘other’ (not specified) levels of grading for their position.

**Figure 2:**
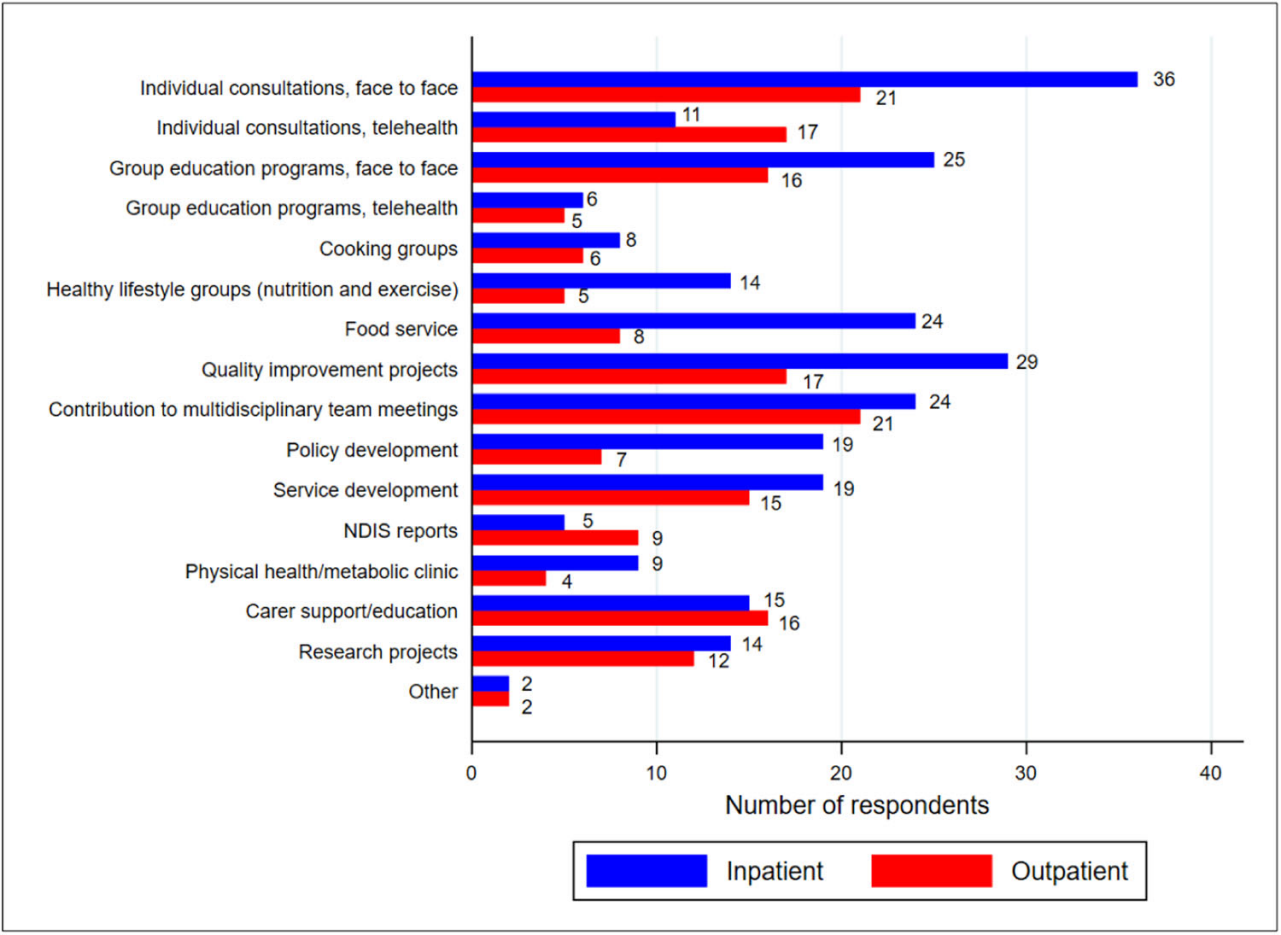
Tasks that dietitian‐respondents (*n* = 48 respondents) complete in their current mental health roles within their respective inpatient (*n* = 36 workplaces) and outpatient (*n* = 23 workplaces) workplace settings. Respondents could select more than one response. NDIS, National Disability Insurance Scheme. *‘Other’ tasks reported by respondents included student supervision and ‘nutrition therapy’.

The top three tasks that respondents reported spending the most time engaged in through their roles were conducting individual consultations (*n* = 47; 98%) group programs via telehealth or face‐to‐face (*n* = 23; 48%) and participating in multidisciplinary team meetings (*n* = 18; 38%); for tasks performed in inpatient and outpatient settings, see Figure 2. Ten respondents reported that a Nutrition and Dietetics Assistant (*n* = 7) or an Allied Health Assistant (*n* = 3) was associated with the dietetic service in their workplace. The range of tasks performed by assistants, reported by respondents, included meal planning and/or ordering meals for patients, meal preparation, nutrition screening and administrative tasks, such as menu printing.

Twenty‐nine respondents reported that nutrition screening or screening for nutrition risks was carried out within their respective workplaces. Screening occurred more often in inpatient settings (*n* = 27 of 29) than in outpatient/community services (*n* = 4 of 29). In inpatient settings, nutrition screening was predominantly completed by nursing staff (*n* = 27) and also by, but less often, allied health staff (*n* = 2) or nutrition/dietitian assistants (*n* = 1), with a similar pattern in outpatients. Nutrition screening in the majority of inpatient settings was conducted using standard malnutrition screening tools with the most common being the Malnutrition Screening Tool^19^ (*n* = 21). In outpatient settings, other measures were used to assess nutritional risk rather than validated malnutrition screening tools.

Ninety percent of respondents (*n* = 43) reported that the dietetic service in their workplace had a process for receiving referrals. Of those receiving referrals, 37 respondents reported that referrals to their service were completed by nursing staff and medical officers, 22 from multidisciplinary team meetings, 20 via self‐referrals and 15 via referrals completed by care coordinators. ‘Other’ referrals, reported by the respondents, included referrals received via an automated nutrition screening system that utilised the Malnutrition Screening Tool, or completed by allied health staff, psychiatrist, mental health nurse, peer support worker, inpatient dietitian referring a patient to an outpatient service, or via family referral. Referrals were most often reported to be received via electronic system format (e.g., electronic medical record; *n* = 29), followed by email (*n* = 22), verbal request (*n* = 22) and in paper form (*n* = 12). Thirty‐two respondents reported that within their service they had a process for triaging referrals. Of these, 25 indicated that a dietitian referral prioritisation protocol or guidelines were used to support triage of service.

### Experience within the dietetic role and with supervision

Within their current workplaces (*n* = 63 workplaces), 24 respondents reported having a dietitian as their direct line manager and 39 reported having a non‐dietetic health professional (e.g., nurse, allied health professional) as their direct line manager. Three‐quarters of the respondents (*n* = 37; 77.1%) reported receiving clinical (*n* = 29) and/or professional (*n* = 25) supervision within their respective roles, with 17 reporting that they received both. For the majority of those receiving clinical supervision, supervision was provided by another dietitian (*n* = 26). Similarly, for those respondents receiving professional supervision, supervision was provided by another dietitian (*n* = 19) and a smaller number received supervision from an Allied Health Professional (*n* = 5). For most respondents (*n* = 21) professional supervision was provided within their organisation, whereas, for nine respondents, supervision was received external to their organisation. The purpose of professional supervision was largely cited as quality improvement (*n* = 17), leadership (*n* = 11), career progression (*n* = 10) and/or management (*n* = 5). With 86% of respondents (*n* = 32 of 37) indicated they were ‘satisfied’ or ‘very satisfied’ with their supervision. Of the 11 respondents not receiving supervision in their current role, the most common reasons reported related to the lack of capacity within the facility or service. Overall, 83% of respondents (*n* = 40 of 48) reported being confident (rated 4 or 5 on five‐point Likert scale) in their current role. Respondents reporting being ‘not confident or unconfident’ had either graduated less than 5 years ago (*n* = 3), had been employed in their current position for 3 years or less (*n* = 6), or were currently receiving no supervision (*n* = 3).

### Dietitian's views on barriers, enablers and challenges to effective service provision

Key themes were identified from the open‐ended questions. The first identified was a lack of funding for dietetic positions within services/facilities (*n* = 30 of 43 respondents). Several respondents highlighted that there was an increasing need for dietitians within services (‘… whilst often recognising the need for dietitian, do not have the finances to expand positions’, R26; ‘Lack of time allocation for dietetics in the mental health service in an increasing area of need’, R15; and ‘… over the last 10–20 years this need has increased …’, R19). As a consequence of the lack of funding, it was suggested that there were ‘minimal senior jobs for dietitians’ (R2) in mental health services, and the lack of funding for dietetic positions led to ‘… inadequate career progression opportunities’ (R2). Respondents commented that it is ‘Difficult to expand roles*…*’ due to a lack of funding within the service, and facilities are often ‘… reluctant to employ more people’ (R10).

Many respondents indicated that additional dietitians would allow ‘more time to complete quality improvement’ activities and to develop the service for ‘continuum of care’. For example, ‘expanding to community services’, ‘facilitate more group work/programs’ and ‘… development and roll out of metabolic monitoring’.

A second theme reported by 26 of the 43 respondents was the suggestion that there is a ‘Misunderstanding of the complexity of our roles and what we (dietitians) can offer to the population therefore when roles get created, they are for insufficient fractions of FTE somewhat due to lack of consultation with dietitians/mental health dietitians in role creation’ (R7). It was suggested there is a ‘lack of knowledge from other health staff’ (R15) and that ‘Dietitians are often overlooked as part of the multidisciplinary team in the management of people with mental illness’ (R46). Furthermore, two respondents considered that the ‘Diet and mental health link is often not considered a concern without malnutrition presentation’ (R23) and that there was a ‘greater emphasis on behavioural risks/self‐harm/mental health status and less emphasis on physical health issues’ (R1) related to nutrition. Several respondents voiced the need for ‘Changing attitudes to nutrition’, ‘Advocating for importance of nutrition’ and ‘Changing protocols’ within mental health services. One respondent commented that ‘Buy in from other professions/patients regarding impact/role of nutrition/dietitians in mental health which impacts nutrition screening compliance and implementation of nutrition intervention’ (R36) was important for increased awareness. As a follow on for effective service provision sufficient staffing, with adequate FTE to fulfil tasks within roles, was considered an important driver by the majority of respondents (*n* = 32 of 45 respondents). This enables dietitians ‘… to see consumers frequently to build rapport prior to jumping straight into nutrition education’ (R39), allows time for ‘… *CPD* [Continuing Professional Development] activities, service review, quality improvement’ (R2) and ‘… time to complete projects or service development, time to collaborate with MDT [multidisciplinary team] and contribute to discussions …’ (R1). More than half the respondents (*n* = 24 of 45 respondents) deemed a ‘multidisciplinary team approach’ as a driver of effective service provision, with collaboration between dietitians and other health professionals, and ‘equal emphasis on all professionals involved …’ (R22). Adequate leadership and supervision (*n* = 13 of 45 respondents), and dedicated workspace with adequate resources (*n* = 12 of 45 respondents) were further drivers identified.

Lack of knowledge, skills and training was identified as a theme to dietitians being employed in mental health services (n = 14 of 43 respondents), with respondents suggesting that ‘More expertise [is required] by clinician to work in mental health’ (R11) and ‘Many dietitians do not have adequate training to support mental health patients’ (R13). Many respondents considered that there is a ‘lack of education in mental health in tertiary education*’* (R31) and ‘… it is lacking in dietetic curriculums at university’ (R46). One respondent commented ‘Ultimately resulting in frequent turnover or employment of new grads in isolating roles without appropriate support’ (R41). It was also suggested, that for many dietitians, there is a ‘Lack of knowledge that it exists as a career option’ (R5). Other examples from participants included greater access to ‘evidenced based guidelines specific to mental health and nutrition’ (R11) and ‘more resources and literature to guide practice’ (R18). Equally, participants indicated a lack of supervision and support (*n* = 8 of 46 respondents) from, for example ‘other dietitians working in mental health’ (R33) or from ‘more senior dietitians specialising in this area’ (R46), and a lack of ‘networking opportunities with dietitians in similar roles’ (R37); as well as a need for more staff or increased hours for dietitians to fulfill tasks within their jobs (*n* = 13 of 42 respondents); and a need for working collaboratively within a multidisciplinary team for the management of patients (*n* = 11 of 42 respondents). ‘Isolation’ and ‘burn out’ were terms commonly expressed in regard to both challenges and barriers. Several respondents (*n* = 8 of 46 respondents) commented that working in mental health ‘can take an emotional toll on the clinician’ (R10) and ‘can be more emotionally taxing than other dietetic fields’ (R46).

Respondents (*n* = 11 of 46 respondents) indicated that it can be challenging to promote dietary change in this ‘complex patient group’ (R12) because individuals often have ‘difficulty engaging in lifestyle change’ (R3), are reluctant to change their diet and ‘… may not be motivated to or adhere with nutritional recommendations’. (R42) One respondent commented many ‘Patients/clients [had a] lack of interest in lifestyle interventions. [They are] just wanting medication and psychology sessions’ (R10). However, it was expressed that the ‘Mental health status of patients may impact whether a patient is appropriate for nutrition intervention/education’ (R36) and that it can be difficult ‘demonstrating effectiveness in change’ (R6). Additionally, several respondents believed there was ‘pressure for weight loss management from other clinicians and doctors when it may not be the most effective approach’ (R6). One respondent commented ‘Approaching nutrition from a perspective of client centred wellness and health (rather than weight loss and deficit) in my experience is more well received by clients and promotes desire to act on change for self‐care’ (R26).

Strategies relating to screening tools were also suggested. This included the implementation of ‘nutrition screening tool specific to mental health population groups’ (R1), the ‘Implementation of screening tool to capture over nutrition earlier’ (R36), the ‘Implementation of a metabolic screening tool’ (R36) and a ‘Dietitian assistant to assist with screening’ (29).

## DISCUSSION

The present study explored dietitian's roles and workforce details, including experience and perspectives, in a sample of Australian dietitians working in a range of mental health settings. This included responses from 48 dietitians working with consumers from across the lifespan, and across a diverse range of mental illnesses. The study identified that dietitians are often working in part time roles with highly varied tasks and responsibilities, which included individual and group consultations, as well as working collaboratively in a multidisciplinary team and alongside allied health assistants. In many settings, nutrition screening was reported as not routinely undertaken and often for malnutrition only. The results highlight the importance of supervision within the workplace for increased confidence in roles for the management of consumers in mental healthcare. There was an expressed need for more dietitian positions in mental healthcare, and increased funding to address staff shortages and provide adequate FTE for dietetic staff to fulfil tasks within their roles to maximise service delivery to consumers. Increased access to evidence‐based resources and training were areas identified as enablers for better service provision.

Participating in multidisciplinary team meetings was a task commonly performed by dietitians and the majority of dietitians reported the collaboration with the multidisciplinary team to be a key driver for effective service provision. However, the lack of understanding regarding the role and value of dietitians in mental health, was perceived to be not only a barrier, but also a challenge for dietitians in providing adequate care. Extending the knowledge of mental healthcare professionals regarding a dietitian's role, was suggested as a strategy to overcome this barrier. Dietitians Australia now includes a mental health role statement^10^ that can support advocacy, and highlights that dietitians as sole practitioners or as members of multidisciplinary teams are an integral part of the management of individuals with mental illness.

Some dietitians in the present study reported being the sole dietitian in their setting working across elements of the nutrition care process,^20^, ^21^ whereas other respondents were part of a dietitian workforce to deliver care. More than half of the dietitians were employed in senior positions, indicating the speciality skill set required in mental health roles; however, many dietitians reported there were limited pathways for development and progression into more senior positions.

The planning and implementation of effective nutrition interventions in mental healthcare requires a valid screening and assessment of an individuals’ nutritional status to identify ‘at‐risk’ patients. In the current analysis, nutritional screening was most often carried out in inpatient settings, with screening not routinely undertaken by dietitians themselves, but more commonly completed by nursing staff. Therefore, engaging the whole team and not just dietitians in training opportunities to upskill for screening patients would allow routine screening to extend beyond malnutrition that may indicate/warrant nutrition intervention, potentially enabling the monitoring of such behaviours as overnutrition and broader disordered eating behaviours. Screening in outpatient settings should be an area of focus in future research and care because it was identified that screening was not conducted as often in outpatient settings when compared to inpatient.

In the present study, the tools or methods used for nutritional screening most often related to malnutrition or methods that detect individuals at risk of undernutrition (e.g., assessing recent poor intake, recent weight loss), rather than overnutrition or other disordered eating behaviours. A systematic review and meta‐analysis of 58 studies reported that the diets of individuals living with severe mental illness were often characterised by lower intakes of nutrient‐dense foods, such as fruits, vegetables and/or fish, and higher intakes of energy‐dense, nutrient‐poor foods.^22^ Furthermore, higher energy and sodium intakes were associated with poorer diet quality and eating patterns.^22^ Currently, there are no validated population specific nutritional screening methods^23^ that assess undernutrition, overnutrition or disordered‐eating behaviours alongside malnutrition in individuals with mental health disorders.

The diversity of the dietitian's role in mental health settings, as highlighted in the results, is largely attributed to the unique needs and challenges in this population group. These include the diversity in nutritional knowledge and varying cognitive abilities, often decreased motivation, and complications related to adverse effects of antipsychotic medications on appetite and metabolic regulation. All of these can not only impact a person's ability for food choices, but also impact abilities for food purchasing, preparation and consumption. Importantly, group education programs were conducted by the majority of dietitians in their day‐to‐day roles and, for many, the running of cooking groups and healthy lifestyle groups that focused on nutrition and exercise was a part of the service provision in their respective roles. Given the higher prevalence of lifestyle diseases (e.g., diabetes, metabolic syndrome, cardiovascular disease, and obesity) among individuals with mental illness,^9^ increasing nutrition literacy and the promotion of healthy behaviours, via group education or individual consultation, is essential to help improve physical health outcomes.

Many dietitian respondents reported that they are not provided with adequate resources and training within the workplace with respect to comprehensively supporting consumers with mental illness. This finding may be attributable to many dietitians being the only dietitian in their setting. This suggests that increased access to nutrition and mental health resources, such as practice guidelines, and more rapid translation of evidence to practice will be essential to support not only dietitians in their roles, but also the broader multidisciplinary team. Furthermore, dietitian respondents suggested that mental health‐specific training across undergraduate and postgraduate levels, as well as continuing professional development, would better prepare student dietitians and practising dietitians for clinical work with consumers with mental illness to potentially reduce barriers such as burn out. Although it was viewed by dietitians that further education and/or training would be beneficial, most dietitians reported being confident in their current roles. This finding supports the importance of clinical or professional supervision because those few who were not confident in their roles reported receiving no supervision. Although mentoring can be valuable, it has been suggested that a more formalised supervision arrangement enables better working practices, improves knowledge and skills, addresses issues in an objective manner, and can minimise stress and burnout for dietitians, particularly those regularly encountering patients with high degrees of psychological distress.^24^

The current survey examined the perceptions of dietitians currently working in a mental health role and included a range of dietitians from diverse clinical experience levels, employment settings and geographical locations within Australia. A limitation is the small sample size, and, because dietitians working exclusively in private practice or eating disorder services were not included in the present study, the results may not be generalisable to all dietitians working in mental health settings. There is a risk of bias with the use of a self‐report survey, particularly self‐selection bias of individuals with a greater willingness to voice their issues or concerns coming forward to participate. It is possible that dietitians not included in the present study may offer differing perspectives to those described. Further research is warranted.

Dietitians, as members of collaborative mental healthcare teams, can improve the health and quality of life of individuals with mental illness. To increase health care delivery and dietitians to work effectively, the present study supports increased financial resources to expand dietetic services in inpatient and outpatient settings, as well as upskilling or expanding mental health content in dietetics training. The development of population specific nutrition screening tools was also highlighted and it will be important to cover broader aspects beyond malnutrition. Advocacy to other health professionals regarding the dietitian's role and capabilities was also identified as an enabler.

## AUTHOR CONTRIBUTIONS

Tracy L. Burrows, Elise Tripodi, Scott B. Teasdale, Janice Plain and Alexandra Harman conceptualised and designed the study. Tracy L. Burrows, Scott B. Teasdale and Elise Tripodi conducted analysis and/or interpretation of the data. Tracy L. Burrows drafted the original manuscript. All authors have critically reviewed its content and have approved the final version submitted for publication.

## CONFLICTS OF INTEREST STATEMENT

The authors declare that there are no conflicts of interest.

## TRANSPARENCY DECLARATION

The lead author affirms that this manuscript is an honest, accurate, and transparent account of the study being reported. The reporting of this work is compliant with STROBE guidelines. The lead author affirms that no important aspects of the study have been omitted and that any discrepancies from the study as planned have been explained.

### PEER REVIEW

The peer review history for this article is available at https://www.webofscience.com/api/gateway/wos/peer-review/10.1111/jhn.13203.
